# Sulfate-Reducing Bacteria Induce Pro-Inflammatory TNF-α and iNOS via PI3K/Akt Pathway in a TLR 2-Dependent Manner

**DOI:** 10.3390/microorganisms12091833

**Published:** 2024-09-05

**Authors:** Sudha B. Singh, Cody A. Braun, Amanda Carroll-Portillo, Cristina N. Coffman, Henry C. Lin

**Affiliations:** 1 Biomedical Research Institute of New Mexico, New Mexico Veterans Affairs (VA) Health Care System, 1501 San Pedro Dr. SE, Albuquerque, NM 87108, USA; sbsingh14@salud.unm.edu (S.B.S.); cody.braun1@va.gov (C.A.B.); ccoffman1@unm.edu (C.N.C.); 2 Division of Gastroenterology and Hepatology, Department of Medicine, University of New Mexico, Albuquerque, NM 87131, USA; acarrol1@unm.edu; 3 Medicine Service, New Mexico Veterans Affairs (VA) Health Care System, 1501 San Pedro Dr. SE, Albuquerque, NM 87108, USA

**Keywords:** *Desulfovibrio vulgaris* (DSV), inducible nitric oxide synthase (iNOS), phosphoinositide 3-kinase (PI3K)/protein kinase B (AKT) (PI3K/AKT), toll-like receptor 2 (TLR 2), tumor necrosis factor-α (TNF-α)

## Abstract

*Desulfovibrio*, resident gut sulfate-reducing bacteria (SRB), are found to overgrow in diseases such as inflammatory bowel disease and Parkinson’s disease. They activate a pro-inflammatory response, suggesting that *Desulfovibrio* may play a causal role in inflammation. Class I phosphoinositide 3-kinase (PI3K)/protein kinase B (AKT) signaling pathway regulates key events in the inflammatory response to infection. Dysfunctional PI3K/Akt signaling is linked to numerous diseases. Bacterial-induced PI3K/Akt pathway may be activated downstream of toll-like receptor (TLR) signaling. Here, we tested the hypothesis that *Desulfovibrio vulgaris* (DSV) may induce tumor necrosis factor alpha (TNF-α) and inducible nitric oxide synthase (iNOS) expression via PI3K/Akt in a TLR 2-dependent manner. RAW 264.7 macrophages were infected with DSV, and protein expression of p-Akt, p-p70S6K, p-NF-κB, p-IkB, TNF-α, and iNOS was measured. We found that DSV induced these proteins in a time-dependent manner. Heat-killed and live DSV, but not bacterial culture supernatant or a probiotic *Lactobacillus plantarum*, significantly caused PI3K/AKT/TNF/iNOS activation. LY294002, a PI3K/Akt signaling inhibitor, and TL2-C29, a TLR 2 antagonist, inhibited DSV-induced PI3K/AKT pathway. Thus, DSV induces pro-inflammatory TNF-α and iNOS via PI3K/Akt pathway in a TLR 2-dependent manner. Taken together, our study identifies a novel mechanism by which SRB such as *Desulfovibrio* may trigger inflammation in diseases associated with SRB overgrowth.

## 1. Introduction

Sulfate-reducing bacteria (SRB) belonging to the genus *Desulfovibrio* are gram-negative motile anaerobic bacteria that are present as minor members of the gut bacterial community in humans [1,2]. These hydrogen sulfide (H_2_S)-producing bacteria exhibit characteristics of a pathobiont that overgrows in the setting of gut microbial dysbiosis such as that observed in diseases including inflammatory bowel disease (IBD), cancer, metabolic syndrome, and neurodegenerative diseases [2,3]. We and others have shown that *Desulfovibrio* activates inflammatory responses such as production of pro-inflammatory cytokines IL-1β, IL-6, IL-8, [4,5] and nitric oxide (NO) [6]. Whether *Desulfovibrio* mediates its pro-inflammatory effects via PI3K/Akt, a major signal transduction pathway critical in orchestrating immune responses, remains unknown. The Class I phosphoinositide 3-kinase (PI3K)/protein kinase B (Akt)/(PI3K/Akt) pathway regulates several key events in the inflammatory response to infection. Inhibitors of the PI3K/Akt pathway have been shown to reduce the severity of inflammation in various experimental models [7,8].

Class I PI3K are heterodimers consisting of a regulatory p85 subunit and a catalytic p110 subunit and are further subdivided into Class IA and IB PI3K [9,10]. Upon activation, PI3K phosphorylates phosphatidylinositol 4,5-bisphosphate (PI (4,5) P2) to form phosphatidylinositol 3,4,5-trisphosphate (PI (3,4,5) P3, usually referred to as PIP3) [11]. PIP3 in turn recruits and binds to the pleckstrin homology (PH) domain of Akt. Akt is activated by Phosphoinositide-Dependent Kinase 1 (PDK1) and PDK2 (mTORC2) at Thr308 and Ser473, respectively [12,13]. Once activated, Akt further orchestrates many downstream processes such as cell proliferation, metabolism, and inflammatory responses, depending upon extracellular stimuli and cell type [12,14]. PI3K/Akt signaling is initiated by binding of receptors such as receptor tyrosine kinases (RTKs), G-coupled protein receptors (GPCR) [10,15] and can also be activated by pathogen-associated molecular patterns (PAMPs) receptors such as Toll-like receptors (TLRs) TLR 2, TLR4, and TLR5 [16,17]. Among these TLRs, TLR4 is activated mainly by bacterial LPS, while TLR5 is activated by flagella [18]. TLR 2, on the other hand, is activated in conjunction with TLR1 or TLR6 by forming heterodimers with these TLRs, which recognize triacylated lipopeptides by TLR 2-TLR1 and diacylated lipopeptide by TLR 2-TLR6 complex [19]. Together these receptor complexes bind a plethora of ligands such as lipoteichoic acid (LTA), lipopeptides, peptidoglycan, zymosan, and other PAMPs from bacteria, fungi, parasites, and viruses [20,21].

In this study, we tested whether *Desulfovibrio* spp. *vulgaris* (DSV) activates the PI3K/Akt pathway and its downstream effectors leading to pro-inflammatory TNF-α and iNOS production. As TLR may activate proinflammatory cytokines in macrophages downstream of the PI3K/Akt pathway in response to bacteria or their products, we also investigated whether DSV-induced PI3K/Akt/TNF/iNOS pathway was dependent on TLR signaling.

## 2. Materials and Methods

### 2.1. Cell Culture and Treatments

RAW 264.7 murine macrophage-like cells (ATCC, Manassas, VA, USA; TIB-71™) were grown in DMEM (Thermo Fisher Scientific, Waltham, MA, USA; 21063-029) + 10% FBS (Thermo Fisher; 26140-079). Cells were grown at 37 °C in a humidified incubator with 5% CO_2_. Prior to the day of infection, 8 × 10^5^ cells were plated in a 6-well plate. The following day, cells were infected with DSV at a multiplicity of infection of 20 (MOI 20) for various times. For drug treatments, cells were incubated with LY294002, a PI3K inhibitor (Cell Signaling Technology, Danvers, MA, USA; 9901) at 10 or 50 µm for 2 h followed by DSV infection for 30 min or 4 h. For TL2-C29 treatment, a TLR 2 antagonist (Invivogen, Waltham, MA, USA; inh-c29), cells were incubated with the inhibitor at 200 μm for 3 h followed by infection with DSV for 4 h.

### 2.2. Bacteria

*Desulfovibrio vulgaris* Hildenborough (ATCC; 29579™) was grown anaerobically for 24 h. in Hungate tubes containing Postgate’s organic liquid medium. Media composition: 10.56 mM Na_2_SO_4_, 13.29 mM MgSO_4_, 4.12 mM L-Cysteine, 0.4% sodium lactate (60% syrup), 0.4% yeast extract, and 0.5% tryptone. Cultures were grown for ~24 h in 5 mL aliquots at 37 °C. *Lactobacillus plantarum* (ATCC; 8014 ™) was grown in Lactobacilli MRS Broth (BD288130) in a 37 °C incubator for ~24 h in 10 mL medium. Bacteria were counted using a Quantom Tx cell counter (Logos Biosystems, Anyang, Republic of Korea).

### 2.3. Western Blotting

Cells were lysed in Lysis buffer (Thermo Fisher Scientific: #87787) containing protease and phosphatase inhibitors (Thermo Fisher Scientific: #1861281) for 20 min on ice. Lysates were centrifuged at 12,000 rpm for 5 min at 4 °C and supernatants were collected. For analysis of secreted TNF-α, cell culture supernatant was precipitated using 9 volumes of chilled ethanol and incubated overnight at −20 °C. The next day, samples were centrifuged at 12,000 rpm for 15 min at 4 °C. Pellets were air-dried and resuspended in PBS. Protein concentration in the samples was determined with Bradford reagent (Bio-Rad Laboratories, Hercules, CA, USA; 5000205). Fifty µg of protein samples were run on SDS-PAGE (4–20% tris-glycine; Thermo Fisher Scientific XP04200BOX) and transferred to nitrocellulose membranes (Bio-Rad; 1620112). Membranes were blocked in 5% milk in PBS (Thermo Fisher; 10010-023) with 0.1% Tween 20 (Sigma, St. Louis, MO, USA; P1379) for 30 min followed by overnight incubation in primary antibodies in a cold room. All antibodies except iNOS were purchased from Cell Signaling Technology: Actin (4970), p-AktS473(4060), p-Akt T308(13038), Akt (9272), p-P70S6K (9205), P70S6K (2708), TNF-α (11948), p-NFκBp65 (3033), and NFκB p65 (4764). iNOS antibody was purchased from Abcam (ab178945). Antibodies were diluted as recommended by the manufacturer. The following day, blots were washed with PBS-T and incubated with secondary antibodies (Cell Signaling Technology: #7074) at room temperature for 1 h (dilution of 1:1000–2000) and developed using enhanced chemiluminescence HRP signal (Thermo Fisher Scientific: 32106, 34577, 37075).

### 2.4. Statistical Analysis

All graphs were generated using Graph Pad Prism 9.4.1. Data are plotted as Mean ± SEM relative to control. Each experiment was conducted at least three independent times. For Western blot analysis, within each independent experiment, three biological replicates were combined to represent one treatment group. For data analysis, comparison between two groups was made using a two-tailed *t*-test. One-way ANOVA with a post-hoc Dunnett’s multiple comparison test was used for comparing the difference between three or more groups. *p* values < 0.05 were considered significant.

## 3. Results

### 3.1. DSV Induced Activation of PI3K/Akt/TNF/iNOS

**Figure 1:**
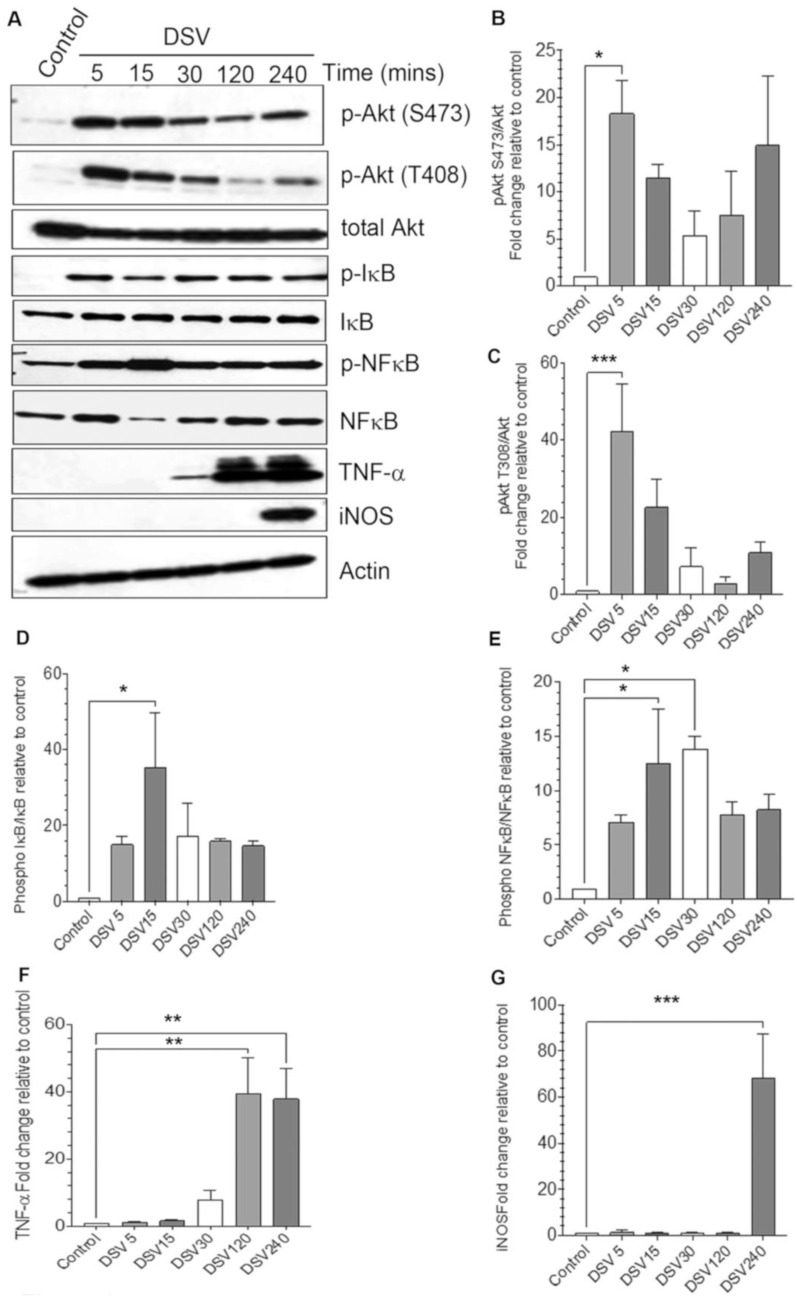
DSV induced activation of PI3K/Akt/TNF pathway in a time-dependent manner. (**A**) RAW 264.7 cells (8 × 10^5^) were infected with DSV at MOI 20 for 5, 15, 30, 120, or 240 min. Cells were lysed and protein lysate was prepared. Fifty μg of protein lysate was separated on SDS-PAGE and analyzed for the expression of p-Akt, total Akt, TNF-α, iNOS, p-IκB, total IκB, p-NFκB p65, and total p65 NFκB by Western blotting. Original blots were cut at different molecular weights to enable probing of as many proteins as possible in the same blot. Actin was used as a loading control. (**B**–**G**) Quantification of Western blots. Blots were quantified with Fiji ImageJ by analyzing the ratio of protein of interest/Actin. For phosphorylated proteins, the ratio of phosphorylated/total protein was calculated. Data represent Mean ± SEM from at least three independent experiments. Values were normalized to control. One-way ANOVA was used to determine the statistical significance. Values were compared to control, with a post-hoc Dunnett’s test. * *p* < 0.05, ** *p* < 0.01, *** *p* < 0.001.

We first tested whether DSV induced PI3K/Akt/TNF and iNOS activation in a time-dependent manner. RAW 264.7 cells were treated with DSV (MOI 20) for various time periods from 5 min to 240 min (Figure 1). Our previous studies have shown that DSV induces optimal effects at MOI 20 [4,22]. Thus, in this study, we used this MOI for all infections. We found that DSV induced phosphorylation of Akt at both Thr 308 and Ser 473 positions (Figure 1A–C) that are activated by PDK1 and PDK2, respectively [23,24], as early as 5 min. Values were significantly higher when compared to control at 5 min post-infection for p-Akt S473 (DSV: 18.33 ± 3.45; Control: 1.00, *p* < 0.05, Figure 1B) and p-Akt T308 (DSV: 42.35 ± 12.30; Control: 1.00, *p* < 0.001, Figure 1C). Levels of p-Akt dropped over time but the trend showed consistently higher levels when compared to control levels. Next, we measured p-IκB and p-Nuclear factor-κB (NFκB) as PI3K/Akt regulates phosphorylation of IκB and transcriptional activity of NFκB [25,26]. We found a significant increase in phospho-IκB levels at 15 min post infection (DSV: 35.41 ± 14.38 vs. Control: 1.00, *p* < 0.05, Figure 1A,D) as well as in phosphorylation of NFκB p65 subunit at 15 and 30 min (DSV 15 min: 12.47 ± 4.97; DSV 30 min: 13.86± 1.130 vs. Control: 1.00, *p* < 0.05, Figure 1A,E) compared to control. Induction of TNF-α occurred as early as 30 min and significantly peaked by 120 min and remained high at 240 min (DSV 2 h: 39.51 ± 10.47; DSV 4 h: 37.92 ± 9.01; Control: 1.00, *p* < 0.01, Figure 1A,F). On the other hand, induction of iNOS was observed only at 4 h (DSV 4 h: 68.20 ± 19.19; Control: 1.00, *p* < 0.001, Figure 1A,G). Thus, DSV induced components of PI3K/Akt pathway and downstream activation of TNF-α and iNOS in a time-dependent manner.

### 3.2. Live and Heat-Killed DSV, but Not Bacterial Culture, Supernatant Significantly Induced PI3K/Akt/TNF/iNOS Pathway

**Figure 2:**
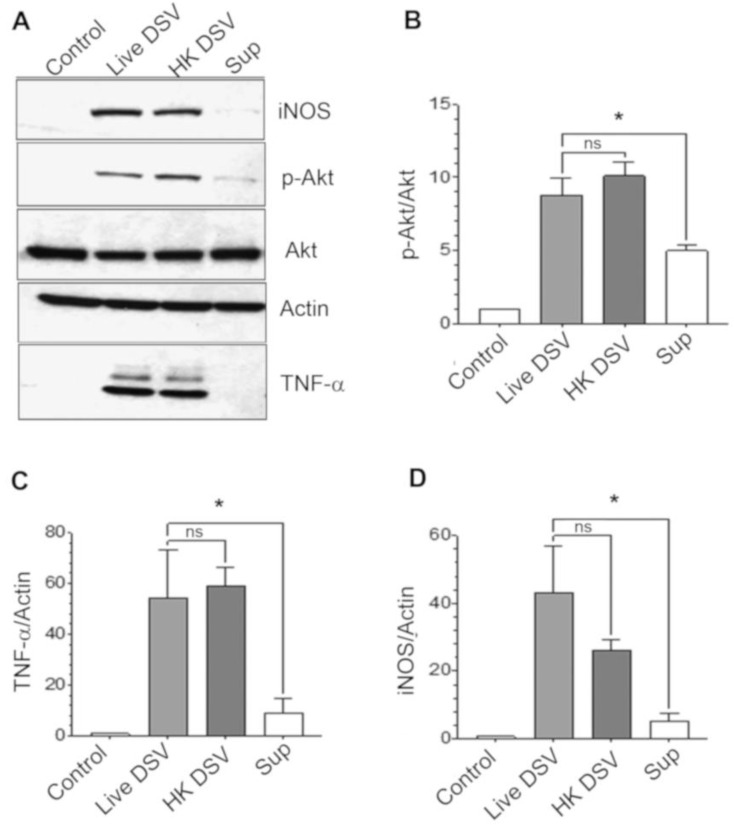
Live and heat-killed DSV, but not bacterial culture supernatant, significantly induced PI3K/Akt/TNF/iNOS pathway. (**A**) RAW 264.7 cells were treated with live or heat-killed (HK) DSV (MOI 20) or bacterial culture supernatant (Sup) for 240 min (4 h). Cells were lysed and protein lysate prepared. Fifty μg of protein lysate was separated on SDS-PAGE and analyzed for p-Akt, total Akt, TNF-α, and iNOS by Western blotting. Actin was used as a loading control. (**B**–**D**) Quantification of Western blots. Blots were quantified with Fiji ImageJ by analyzing the ratio of protein of interest/Actin. For phosphorylated Akt, the ratio of p-Akt/total Akt was calculated. Data represent Mean ± SEM from at least three independent experiments. Values were normalized to control. One-way ANOVA was used to determine the statistical significance. Values were compared to live DSV. * *p* < 0.05, ns (non-significant).

Next, we compared the effects of live DSV with heat-killed DSV and with the culture supernatant of DSV on PI3K/Akt/TNF/iNOS pathway (Figure 2). For generating heat-killed bacteria (HK), DSV were autoclaved and an equal volume of bacteria was added to the cells as live DSV. In the case of culture supernatant (Sup), 1 mL bacterial culture was centrifuged to pellet the bacteria. Sup was further passed through a 0.2 µ filter to remove any remaining bacteria. Sixty μL of sup, equivalent to the volume of resuspended bacteria at MOI 20, was applied to the cells. We found that HK bacteria were equally efficient in inducing p-Akt compared to live bacteria (Live DSV: 8.79 ± 1.15 vs. HK: 10.12 ± 0.91, *p* > 0.05). However, levels of p-Akt were significantly lower in cells treated with Sup in comparison to live DSV (Live DSV: 8.79 ± 1.15; Sup: 4.99 ± 0.33; *p* < 0.05, Figure 2A,B). This trend was reflected in TNF-α expression where HK induced a comparable amount of the protein when compared to DSV but much lower induction of TNF-α by Sup (Live DSV: 54.37 ± 18.62; HK: 59.10 ± 7.28; *p* > 0.05; Sup: 9.29 ± 5.41, *p* < 0.01 compared to DSV, Figure 2A,C). Similarly, HK bacteria induced iNOS levels comparable to DSV but in cells treated with culture sup, much lower levels of iNOS were induced (DSV: 22.37 ± 3.44; HK: 20.16 ± 3.54; *p* > 0.05 compared to DSV; Sup: 5.51 ± 2.62, *p* < 0.01 compared to DSV, Figure 2D). These results suggest that a structural component(s) of DSV rather than the metabolically active DSV is responsible for inducing PI3K/Akt/TNF/iNOS pathway in macrophages. The structural component(s) responsible for these effects of DSV may be an integral part of the bacteria or may be secreted in lower levels, based on our findings with milder effects of Sup when compared to live and HK bacteria.

### 3.3. Comparison of Effects of DSV and Lactobacillus plantarum on PI3K/Akt/TNF/iNOS Activation

**Figure 3:**
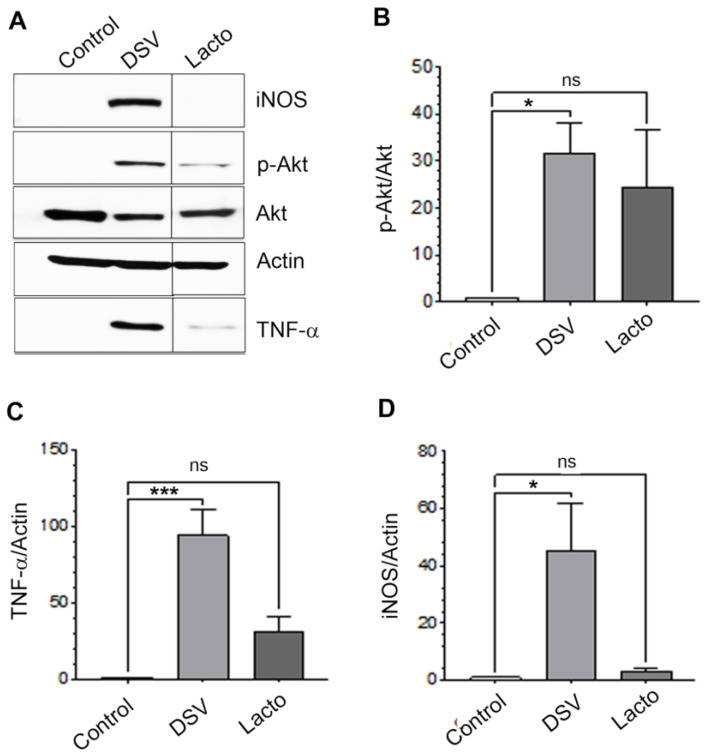
Comparison of effects of DSV and *Lactobacillus plantarum* on PI3K/Akt/TNF/iNOS pathway. (**A**) RAW 264.7 cells were treated with DSV or Lacto (MOI 20) for 4 h. Cells were lysed and protein lysate prepared. Fifty μg of protein lysate was separated on SDS-PAGE and analyzed for p-Akt, total Akt, TNF-α, and iNOS by Western blotting. Lanes were spliced in the original blot and combined. Actin was used as a loading control. (**B**–**D**) Quantification of Western blots. Blots were quantified with ImageJ by analyzing the ratio of protein of interest/Actin. For phosphorylated Akt, the ratio of p-Akt/total Akt was calculated. Data represent Mean ± SEM from at least three independent experiments. Values were normalized and compared to Control. One-way ANOVA was used to determine the statistical significance. * *p* < 0.05, *** *p* < 0.001, ns (non-significant).

Next, we tested whether probiotic bacteria *L. plantarum* induced PI3K/Akt/TNF/iNOS pathway and compared its effects with those of DSV. Cells were treated with *L. plantarum* (Lacto) for 4 h at MOI 20 (Figure 3). As expected, DSV caused a significant increase in p-Akt when compared to control (DSV: 31.75 ± 6.54; Control: 1.00, *p* < 0.05). Interestingly, Lacto also induced p-Akt but the effects were milder than DSV and the levels were found to be statistically insignificant compared to control (Lacto: 24.37 ± 12.50; Control: 1.00, *p* > 0.05, Figure 3A,B). We also found that Lacto caused an increase in TNF-α albeit to a much lesser extent than DSV (DSV: 101.5 ± 20.63; Control 1.00, *p* < 0.001) and these effects were also insignificant when compared to control (Lacto: 22.65 ± 9.187 vs. Control: 1.00, *p* > 0.05, Figure 3A,C). Lacto failed to significantly induce iNOS expression (Lacto: 3.153 ± 0.9706; Control: 1.00, *p* > 0.05, Figure 3A,D) whereas DSV caused a drastic increase in iNOS compared to Control (DSV: 45.45 ± 16.27; Control: 1.00, *p* < 0.05). These findings suggest that these two bacteria diversify in eliciting cellular immune response downstream of PI3K/Akt and cells may generate different responses to a probiotic Lacto when compared to an opportunistic pathobiont DSV. These findings also support the notion that PI3K/Akt can be activated by both beneficial and harmful bacteria and the outcome may be determined by the type of bacteria and by host cells.

### 3.4. LY294002 Inhibited DSV-Induced Activation of PI3K/Akt/TNF Pathway

**Figure 4:**
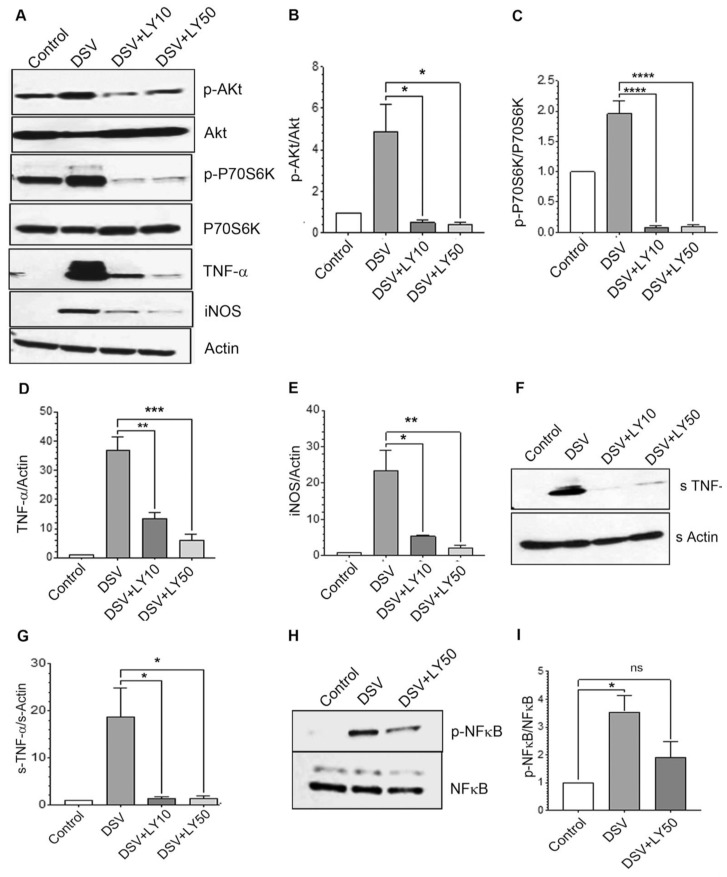
LY294002 inhibited DSV-induced activation of PI3K/Akt/TNF pathway. (**A**) RAW 264.7 cells were treated with LY at either 10 or 50 μM for 2 h before infection with DSV for 4 h. Cells were lysed and protein lysate prepared. Fifty μg of protein lysate was separated on SDS-PAGE and analyzed for p-Akt, total Akt, p-P70S6K, P70S6K, TNF-α, and iNOS, by Western blotting. Actin was used as a loading control. (**B**–**E**) Quantification of Western blots. (**F**) Post-infection, cell culture supernatant was collected and proteins were precipitated using ethanol precipitation. Proteins were resuspended in PBS and 50 μg was loaded on SDS-PAGE. Secreted TNF-α (s-TNF-α) was detected using anti-TNF-α antibody. Actin was used as a loading control. (**G**) Quantification of s-TNF-α. (**H**) Cells were pretreated with or without LY 50 μM followed by infection with DSV for 30 min and protein samples were analyzed for p-p65 NFκB and total NFκB. (**I**) Blots were quantified with ImageJ by analyzing the ratio of p-NFκB/NFκB. Values were normalized and compared to control. Data represent Mean ± SEM from at least three independent experiments. One-way ANOVA was used to determine the statistical significance, with a post-hoc Dunnett’s test. * *p* < 0.05, ** *p* < 0.01, *** *p* < 0.001, **** *p* < 0.0001, ns (non-significant).

LY294002 (LY) is a well-known inhibitor of class I PI3K pathway [27]. To investigate whether DSV induced classical PI3K/Akt pathway that was responsible for upregulating proinflammatory TNF-α and iNOS proteins, we pre-treated the cells with LY (10 or 50 μM) for 2 h before DSV challenge for 4 h (Figure 4). We found that LY inhibited DSV-induced Akt phosphorylation (DSV: 4.88 ± 1.31; DSV + LY10: 0.52 ± 0.11; DSV + LY50: 0.40 ± 0.09, *p* < 0.05 compared to DSV, Figure 4A,B). P70S6K is another effector that is activated downstream of PI3K/Akt via phosphorylation by mTOR complex 1 (mTORC1) [28]. LY inhibited DSV-induced p-P70S6K levels (DSV: 1.96 ± 0.20; DSV + LY10: 0.081 ± 0.026; DSV + LY50: 0.088 ± 0.029, *p* < 0.0001 compared to DSV, Figure 4A,C). We found that cellular TNF-α expression was elevated in the presence of DSV and LY significantly inhibited this effect (DSV: 37.01 ± 4.28; DSV + LY10: 13.42 ± 2.13, *p* < 0.01; DSV + LY50: 6.044 ± 2.139, *p* < 0.001 compared to DSV, Figure 4A,D). LY also inhibited DSV-induced iNOS expression (DSV: 23.54 ± 5.39; DSV + LY10: 5.45 ± 0.17, *p* < 0.05; DSV + LY50: 2.28 ± 0.58, *p* < 0.01, Figure 4A,E). Similarly, levels of DSV-induced secreted TNF-α were inhibited by LY (DSV: 18.73 ± 6.21; DSV + LY10: 1.45 ± 0.24; DSV + LY50: 1.34 ± 0.57, *p* < 0.05 compared to DSV, Figure 4F,G). Next, we checked whether LY inhibited DSV-induced activation of NFκB by measuring the expression of phospho-P65 subunit of NFκB (Figure 4H,I). DSV treatment caused a significant increase in p-p65 when compared to Control (DSV: 3.54 ± 0.59; Control: 1.00, *p* < 0.05). However, prior treatment of cells with LY prevented these effects of DSV (DSV + LY50: 1.91 ± 0.54; Control: 1.00, *p* > 0.05). These results suggest that DSV induced classical PI3K/Akt pathway that was responsible for the downstream induction of proinflammatory TNF-α as well as iNOS.

### 3.5. Activation of PI3K/Akt Pathway by DSV Was Dependent on TLR 2 Signaling

**Figure 5:**
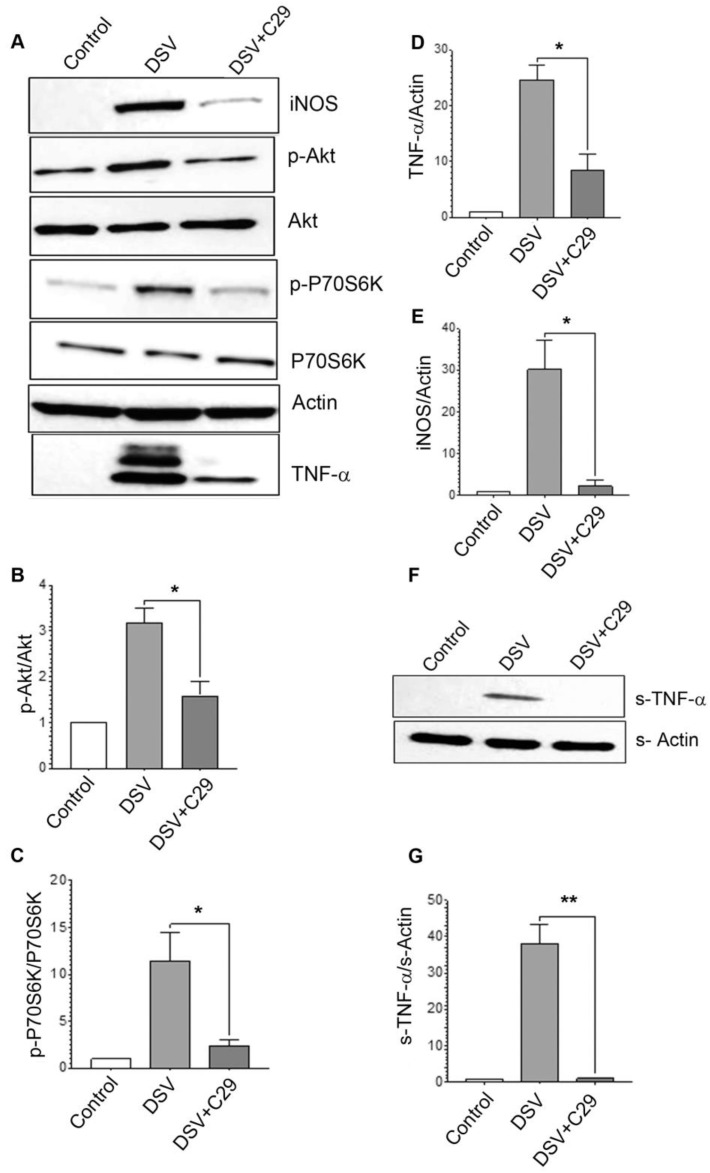
Activation of PI3K/Akt pathway by DSV was dependent on TLR 2 signaling (**A**) RAW 264.7 cells were treated with TLR 2 signaling inhibitor C29 at 200 μM for 3 h before infection with DSV for 4 h. Cells were lysed and protein lysate prepared. Fifty μg of protein lysate was separated on SDS-PAGE and analyzed for p-Akt, total Akt, p-P70S6K, P70S6K TNF-α, and iNOS by Western blotting. Actin was used as a loading control. (**B**–**E**) Quantification of Western blots. (**F**) Post-infection, cell culture supernatant was collected and proteins were precipitated using ethanol precipitation. Proteins were resuspended in PBS and 50 μg was loaded on SDS-PAGE. Secreted TNF-α (s-TNF-α) was detected using anti-TNF-α antibody. (**G**) Blots were quantified with ImageJ by analyzing the ratio of protein of interest/Actin. For phosphorylated proteins, the ratio of phosphorylated/total protein was calculated. Data represent Mean ± SEM from at least three independent experiments. Values were normalized to control. A two-tailed *t*-test was used to compare DSV and DSV + C29. * *p* < 0.05, ** *p* < 0.01.

The PI3K/Akt pathway is activated downstream of receptors such as tyrosine kinase (RTK) and G-protein-coupled receptors, and also by Toll-Like Receptors (TLRs), often observed in cases of infections with various bacteria [29]. Thus, we treated the cells with different TLR antagonists to test the involvement of TLR 2, TLR 4 or TLR 5 in DSV-mediated activation of PI3K/Akt pathway. We found that neither TLR4 antagonist TLR-IN-C34 [30,31] nor TLR5 antagonist TH1020 [32] had any discernible effect on DSV-induced PI3K/Akt signaling. However, pretreatment of cells with TLR 2 inhibitor, TLR-C29 (C29) significantly inhibited DSV-induced effects (Figure 5). We found that DSV-induced phosphorylation of AKT was inhibited by C29 (DSV: 3.18 ± 0.30; DSV + C29: 1.58 ± 0.29, *p* < 0.05, Figure 5A,B). Similarly, expression of p-P70S6K, an effector downstream of Akt, was also upregulated by DSV and was significantly inhibited by C29 (DSV: 11.48 ± 3.05; DSV + C29: 2.39 ± 0.69, *p* < 0.05, Figure 5A,C). Levels of cellular TNF-α protein were also upregulated by DSV and inhibited in the presence of C29 (DSV: 24.67 ± 2.51; DSV + C29: 8.57 ± 2.81, *p* < 0.05, Figure 5A,D). Additionally, DSV-induced iNOS protein expression was significantly inhibited by C29 (DSV: 30.25 ± 6.82; DSV + C29: 2.32 ± 1.48, *p* < 0.05, Figure 5A,E). Levels of DSV-induced secreted TNF-α were also significantly inhibited by C29 (DSV: 38.09 ± 5.188; DSV + C29: 1.103 ± 0.03408, *p* < 0.01, Figure 5F,G). As PI3K/Akt is classically activated by a vast number of stimuli through receptor tyrosine kinases (RTKs), we tested whether DSV-induced PI3K/Akt/TNF/iNOS pathway was dependent on RTK-mediated signaling by using tryphostin AS1478 as an antagonist to EGFR signaling, a common RTK. We did not observe inhibition of the DSV-induced PI3K/Akt activation by tryphostin, suggesting that this RTK was not involved in DSV-mediated effects. Whether other RTKs are activated in response to DSV remains to be determined. Taken together, our results suggest that DSV specifically engages TLR 2 to mediate its pro-inflammatory effects via PI3K/Akt pathway.

## 4. Discussion

*Desulfovibrio* are SRB that are present as minor members of the resident gut bacterial community and are mainly recognized for their production of hydrogen sulfide (H_2_S) as a byproduct of their metabolism. An increase in *Desulfovibrio* density has been reported in many diseases including IBD, metabolic syndrome, cancer, autism, and Parkinson’s disease [2]. While the mechanisms underlying such associations remain largely unknown, few studies have suggested the causal role of *Desulfovibrio* in the pathogenesis of these diseases using various in vivo and in vitro models [2]. As such, our laboratory has previously identified various cellular pathways that are affected by *Desulfovibrio* spp. *vulgaris* such as increased intestinal barrier permeability, decreased lysozyme production (a crucial anti-microbial protein), and increased pro-inflammatory Notch signaling pathway [4,22,33], effects that could contribute to disease development. In this study, we uncover yet another layer of *Desulfovibrio* function that may be responsible for pathogenesis of diseases and which may also act in concert with other known DSV-induced pathways to contribute to disease development. We found that *Desulfovibrio vulgaris* (DSV) induced protein expression of pro-inflammatory TNF-α and iNOS. While activation of immune response by bacteria is an important cellular defense mechanism to eliminate pathogens, uncontrolled immune response is harmful and can lead to inflammation that underlies many acute and chronic diseases such as IBD and sepsis. It is possible that *Desulfovibrio* overgrowth, as seen in conditions such as IBD, may actually play a causal role in the development of inflammatory diseases by activating a proinflammatory immune response.

We found that DSV-induced TNF-α and iNOS expression was dependent on PI3K/Akt pathway as inhibition of PI3K by LY294002 abrogated these effects. PI3K/Akt pathway is a major signal transduction pathway involved in many fundamental cellular processes such as differentiation, survival, apoptosis, transcription, translation, proliferation, as well as immune responses. PI3K/Akt cascade is activated by vast environmental stimuli via several cell surface receptors and which orchestrate a plethora of downstream pathways via diverse effector molecules. Dysfunction of this pathway has been linked to many diseases such as metabolic diseases, cancer, neurodegenerative diseases, vascular diseases, infections, and inflammatory diseases [10,34]. While it is involved in activating a pro-inflammatory response in some infections [35], in others it may play a protective anti-inflammatory role [36,37]. Thus, the role of PI3K/Akt in infection and inflammation is multifaceted and diverse.

In addition to our findings with *D. vulgaris* as a mediator of inflammatory response, other species of *Desulfovibrio* such as *D. piger*, *D. desulfuricans*, or *D. fairfieldensis* have also been shown to induce inflammatory outcomes [2].

In the context of resident gut bacteria, pathobionts such as *Fusobacterium nucleatum* which are implicated in disease development in IBD, esophageal cancer, and colorectal cancer [38,39], have also been found to activate PI3K/Akt pathway [40,41]. In contrast, another gut bacterium *Akkermansia muciniphila* secretes threonyl-tRNA synthetase which elicits anti-inflammatory effects such as IL-10 production in a TLR 2/PI3K/Akt pathway-dependent manner [42]. However, the opposite effects of the same bacteria are seen in a study where *Akkermansia muciniphila* and its metabolites butyric acid and deoxycholic acid inhibited PI3K/Akt pathway to protect against the harmful effects of *Campylobacter jejuni* [43]. Triggering of PI3K/Akt pathway is also observed in the case of probiotics. In this study, we found that infection of RAW 264.7 macrophages with *L. plantarum* induced PI3K/Akt expression, but only mildly induced TNF-α and iNOS activation was negligible in the presence of these bacteria when compared to DSV. Our findings are partly in accord with other reports showing that *L. plantarum* induced M1 type phenotype in macrophages via TLR 2/NFκB signaling, and increased bactericidal activities against *Salmonella* by activating TLR 2/NF-kB signaling pathway [44]. Similarly, *Lactobacillus rhamnosus* can induce M1 phenotype in macrophages to produce cytokines such as IL-1β, IL-10, and TNF-α in a TLR-2/MyD88/MAPK dependent manner as a way to defend against pathogens [45]. Thus, it is possible that probiotics may elicit a “healthy” immune response that is important in combating pathogens as seen in these cases. On the other hand, the probiotic *Lactobacillus paracasei* induces M2 phenotype that is associated with an anti-inflammatory response [46]. Thus, these overall disparate outcomes of PI3K/Akt activation may be dictated by the strain of the bacteria, their metabolic products, type of host cell, cell surface receptors, and the overall disease context.

PI3K/Akt signaling has been vastly studied in the context of metabolism and differentiation downstream of growth factor receptors such as PDGF receptor (PDGFR) and epidermal growth factor receptor (EGFR), insulin-like growth factor receptor (IGFR), and insulin receptor (INSR), RAS, and G-protein coupled receptor, depending upon specific isoforms [10]. However, PI3K/Akt pathway is also known to be activated in a toll-like receptor manner [47,48]. Toll-like receptors (TLRs) are transmembrane receptors that recognize a variety of pathogen-associated molecular patterns (PAMPs) [20,49,50] and are central players in innate immune responses against various bacterial and viral agents [51,52]. TLRs other than TLR3 are known to activate NFκB signaling and inflammatory cytokine production in a MyD88-dependent manner [53]. Studies have shown that p85 subunit of PI3K binds to the cytosolic domain of TLR 2 that contains PI3K binding motif (YXXM) [16,54,55]. TLRs activate PI3K/Akt pathway and further orchestrate downstream effects that are dictated by the type of stimuli [16,17,47,55], Engagement of TLR 2, 4, and 5 with bacterial components leads to pro- or anti-inflammatory responses [56]. Data support the role of LPS from gram-negative bacteria in mediating these effects [57,58]. Similarly, TLR5 is activated by bacterial flagella and activates pro-inflammatory PI3K/Akt pathway [59,60]. While DSV expresses LPS, a major TLR4 ligand [61], we did not see inhibition of DSV-induced PI3K/Akt/TNF/iNOS activation in the presence of TLR4 antagonist. This is in agreement with our previous study where inhibition of TLR 4 also failed to prevent DSV-induced pro-inflammatory Notch signaling pathway [4], suggesting that TLR4 is not involved in the inflammatory immune response elicited by DSV. Additionally, we failed to see inhibition of this pathway in the presence of TLR5 antagonist that inhibits TLR5 activation by flagella [62], suggesting that DSV flagella were not involved in TLR activation downstream of DSV. Moreover, we did not see inhibition of DSV effects by an EGFR inhibitor suggesting that this receptor is not involved. However, it does not rule out involvement of other RTKs that may be triggered by DSV. Further studies are needed to investigate whether other RTKs are involved in this process.

In contrast to TLR4 and TLR5, we observed a significant inhibition of DSV-mediated activation of PI3K/Akt/TNF/iNOS pathway in the presence of C29, a potent antagonist of TLR 2/1 and TLR 2/6 signaling [63] that has been shown to prevent TNF-α production by bacteriocin [64]. While TLR 2 is mostly known to be activated in response to gram-positive bacteria, there are reports of TLR 2 activation in response to gram-negative bacteria such as *Porphyromonas gingivalis* [65,66] *Treponema pallidum* [35], and *Vibrio cholerae* [67]. Which component of DSV induces TLR 2 signaling remains to be determined. Some studies have highlighted the role of hydrogen sulfide in activating TLR signaling [68]. Interestingly, a decreased level of H_2_S and *Desulfovibrio* spp. in the colon/feces was found in TLR 2^−/−^ mice while it increased in TLR4^−/−^ mice, suggesting an intimate link between SRB and TLR 2 in vivo [69]. Moreover, higher expression of TLR 2 has been found in IBD patients [70,71]. Additionally, higher levels of fecal H_2_S are observed in IBD patients [72]. These studies suggest a link between SRB, H_2_S, and TLR 2 signaling. Whether or not H_2_S produced by DSV activates this pathway remains to be tested. Further, dissecting whether DSV involves TLR1/2 or TLR 2/6 complex signaling may shed light on the nature of the ligand in DSV that activates the TLR 2 PI3K/Akt/TNF/iNOS signaling pathway.

Induction of TNF-α and iNOS by DSV is physiologically relevant as high levels of these proinflammatory mediators and their downstream products such as reactive nitrogen species are seen in inflammatory diseases such as IBD [73,74]. In fact, anti-TNF-α antibodies are considered as therapeutic avenues for managing IBD since TNF-α is the main inflammatory mediator in these diseases [75,76]. TNF-α can further activate NFκB and proinflammatory PI3K/Akt pathway [77,78]. Thus, production and secretion of TNF-α by DSV-infected cells can initiate a PI3K/Akt cascade in uninfected cells further amplifying the inflammatory events.

**Figure 6:**
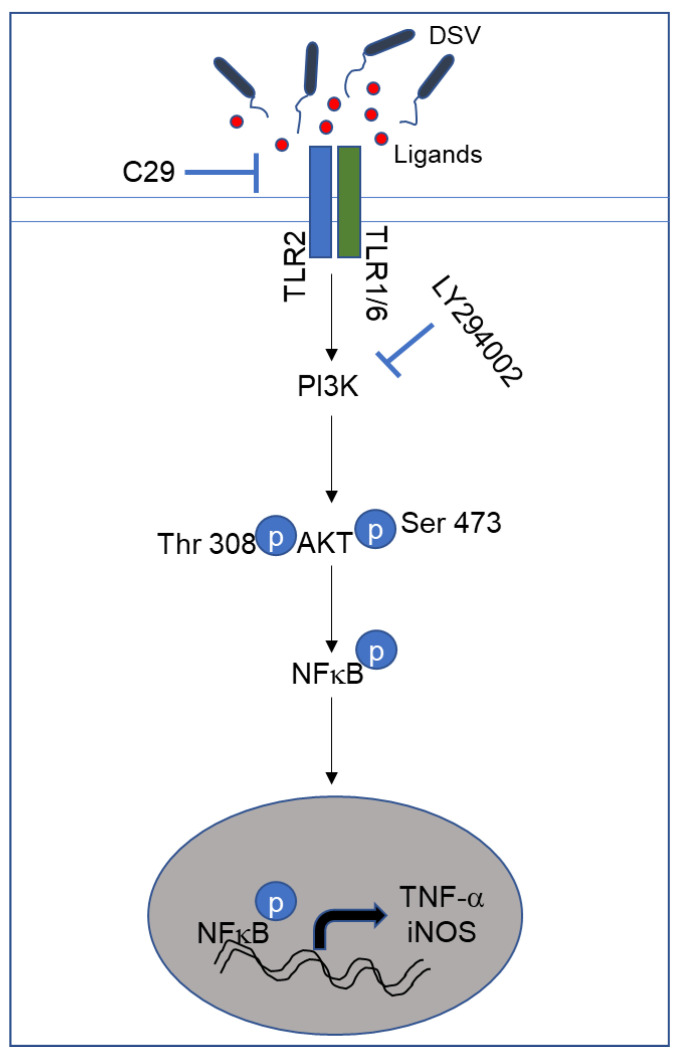
Schematic representation of activation of PI3K/Akt pathway by DSV. DSV activates TLR 2 signaling which is inhibited by C29 (TL2-C29). TLR 2-mediated intracellular signaling occurs via formation of heterodimers of TLR 2 with TLR 1 or TLR 6 (TLR 1/6) that allows recognition of diverse sets of pathogen-associated patterns. TLR 2 activation further leads to the activation of PI3K/Akt as indicated by phosphorylation of Akt at Thr308 and Ser473 positions which is required for a complete activation of Akt. This step is inhibited by PI3K inhibitor LY294002. Once activated, Akt further induces transcription factor NFκB. Upon activation, NFκB induces expression of proinflammatory TNF-α and iNOS.

Our discovery of DSV-induced iNOS expression supports previous findings where DSV induced nitrite production in RAW 264.7 cells [6]. The role of iNOS in inflammation is dichotomous, being both harmful and beneficial to the host [79]. iNOS activation in immune cells occurs in response to infections, leading to increased production of nitric oxide (NO) that is responsible for killing and limiting the infectious agents [80]. However, accumulation of iNOS and NO as seen in IBD may have opposite effects, leading to chronic inflammation in these diseases. As iNOS is capable of producing NO over days [73,81], in the setting of *Desulfovibrio* bloom in inflammatory conditions with dysfunctional immune response, this may lead to chronic accumulation of NO production which can further exacerbate inflammation. Moreover, TNF-α treatment itself can lead to increased production of reactive nitrogen intermediates [82,83]. DSV bloom is also observed in neurodegenerative diseases such as Parkinson’s disease. Gut microbes communicate with the brain via the gut–brain axis, and gut microbial dysbiosis, which leads to leaky gut and inflammation, has been recognized as the basis of development of neurodegenerative diseases [84,85]. As neuroinflammation mediated by the PI3K/akt pathway in neuronal immune cells such as microglia is an underlying pathological mechanism of disease development [86], our findings of DSV-induced inflammatory PI3K/Akt pathway in immune cells raises an important question whether the same mechanism is responsible for neuroinflammation that underlies PD in response to DSV. It will be interesting to study whether and how DSV contributes to neuroinflammation. As such, the inflammatory as well as protective role of PI3K/Akt in PD has been demonstrated in various experimental conditions where the role of PI3K/Akt as a potential therapeutic target is discussed [87,88,89]. Whether or not PI3K/Akt pathway induced by DSV in immune cells contributes in neuro-inflammation and further disease progression remains to be discovered. In addition, it is shown that IBD presents a risk factor for developing PD [90,91]. Moreover, several studies have identified gut bacteria or their bacterial products that may trigger inflammation in the gut as an early onset of PD [92]. Thus, the mechanism of how DSV-induced proinflammatory PI3K/Akt pathway may contribute to diseases of gut-brain axis such as PD remains a subject of future research. Taken together, results from our study suggest that production of TNF-α and iNOS in response to DSV may be responsible in part for inflammation in diseases such as IBD where *Desulfovibrio* overgrow. Figure 6 summarizes our findings in this study.

## 5. Conclusions

Our results identify a novel pathway by which SRB such as *Desulfovibrio* may induce inflammatory events and may explain the mechanisms underlying the association between a DSV bloom and inflammatory diseases. Our study also identifies novel targets that may be helpful in developing therapeutic avenues to treat diseases associated with DSV overgrowth.

## Data Availability

The authors confirm that the data supporting the findings of this study are available within the article.

## Abbreviations

AKT/PKB: protein kinase B; DSV: *Desulfovibrio vulgaris*; H_2_S: hydrogen sulfide; iNOS: inducible nitric oxide synthase; IBD: inflammatory bowel disease; IL-1β: interleukin 1-beta; MOI: multiplicity of infection; NFκB: *Nuclear factor kappa B;* PD: Parkinson’s disease; PAMP: pathogen-associated molecular patterns; PI3K: Phosphoinositide 3-kinase; RTK: receptor tyrosine kinase; SRB: sulfate-reducing bacteria; TLR 2: toll-like receptor 2; TNF-α: tumor necrosis factor-alpha.
